# A Holistic Approach to Chemical Mixtures in the Canadian Environment

**DOI:** 10.1021/acs.est.6c03449

**Published:** 2026-05-29

**Authors:** L. Mark Hewitt, Alicia Berthiaume, Emmanuelle Caron, Zhen Cheng, Rebecca L. Dalton, Tom Harner, Robert J. Letcher, Sylvie Poirier Larabie, Jacob Mastin, Stacey A. Robinson, Marie-Claude Sauve, Tristan A. Smythe, Heather Steckley, Geneviève Tardif

**Affiliations:** † Science and Technology Branch, 6347 Environment and Climate Change Canada, 867 Lakeshore Road, Burlington, Ontario L7S 1A1, Canada; ‡ Science and Technology Branch, 6347 Environment and Climate Change Canada, 351 Boulevard Saint-Joseph, Gatineau, Quebec K1A 0H3, Canada; § Science and Technology Branch, 6347 Environment and Climate Change Canada, 4905 Dufferin Street, Toronto, Ontario M3H 5T4, Canada; ∥ Science and Technology Branch, Environment and Climate Change Canada, Carleton University, Ottawa, Ontario K1A 0H3, Canada

**Keywords:** environmental chemical mixtures, exposure pathways, cumulative effects, risk assessment

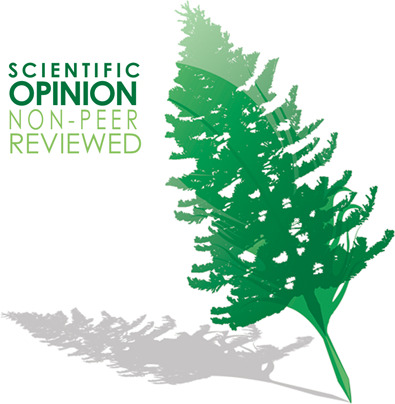

## Graphical abstract

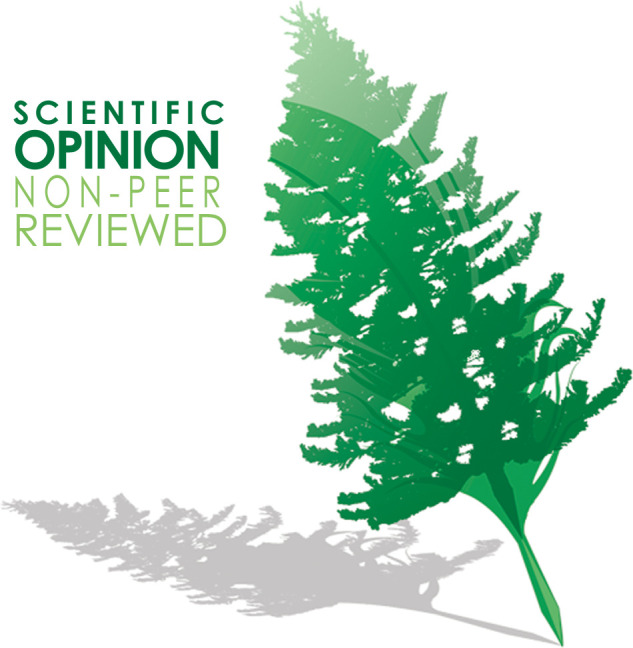

Canada is piloting a new, integrated, regional approach to understanding and managing real-world environmental chemical mixtures under the Canadian *Environmental Protection Act*, 1999 (CEPA), recently modernized through the *Strengthening Environmental Protection for a Healthier Canada Act* in 2023. The Integrated Chemical Mixtures Project (ICMP) encompasses activities in two regions in the south of the Province of Ontario, the City of Brantford/Grand River (mixed inputs, e.g., urban and agricultural land uses) and the City of Sarnia/St. Clair River (heavily industrialized), to generate data on mixture compositions, exposure pathways, and cumulative effects, in direct collaboration with regulatory scientists, all levels of government, environmental organizations, and Indigenous partners (Figure 1). This project provides the conceptual and methodological foundation for future national activities under CEPA.

**Figure 1:**
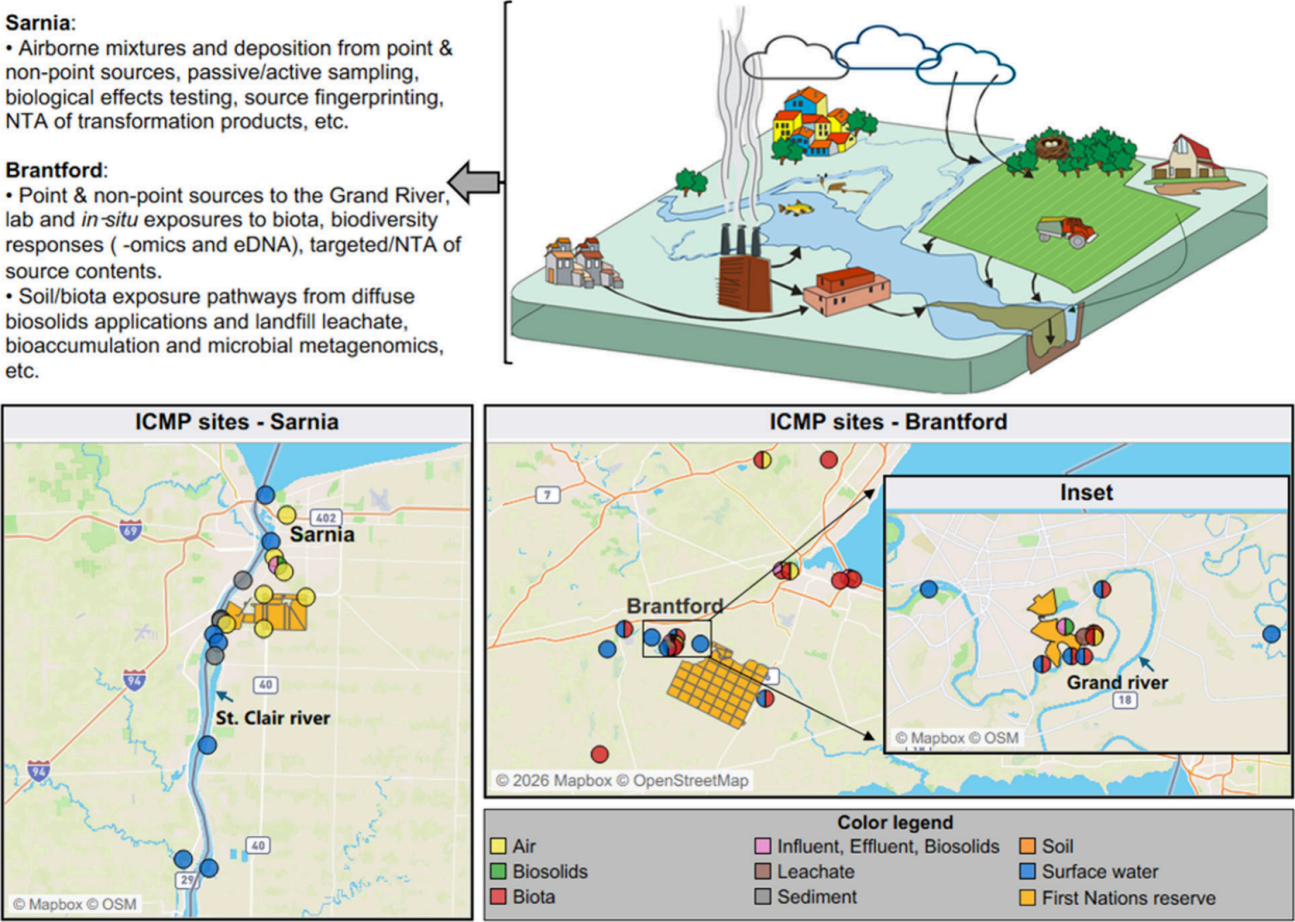
Examples of mixture exposure pathways and sampling sites investigated by the ICMP in Brantford and Sarnia, Ontario, Canada.

## What Do We Mean by Chemical Mixtures in the Environment?

Real-world complex mixtures arise from multiple sources across environmental compartments and dynamically fluctuate across space and time in their composition. Chemical mixtures entering and found in the environment can have single or multiple origins that can include anthropogenic point (e.g., stack) and nonpoint (e.g., urban runoff) sources, as well as natural sources (e.g., forest fires). Mixtures may exert detrimental and complex ecotoxicological effects on ecosystems, wildlife, and human health.^1^

## What Is Being Done about the Risks Posed by Mixtures?

In Canada and other jurisdictions, regulatory programs have sought to mitigate the risks posed by prioritized individual substances or substance groups. Notwithstanding the success of these programs, complex mixtures as they occur in real-world scenarios have not typically been directly addressed. Internationally, the potential for mixture risk has been recognized for more than a decade by organizations, including the World Health Organization^2^ and the European Chemicals Strategy for Sustainability, where mixture allocation factors are being considered in an upcoming revision of Registration, Evaluation, Authorization, and Restriction of Chemicals (REACH).^3^ Collaborative European initiatives (e.g., SOLUTIONS) call for consistent mixture assessment concepts, integration across human toxicology and ecotoxicology, and regional prioritization.^4^ In Canada, regulatory programs are undergoing significant evolution, with assessments increasingly required to consider available information about the cumulative effects of substances. To support this transition, alternative frameworks, including the ICMP, are currently being evaluated.

## Charting a Path Forward: A Holistic Canadian Project

Chemical mixture dynamics and complexities necessitate innovative new approaches to assess and manage identified risks. Through its activities, the ICMP is generating a field-anchored evidence base of mixture compositions, sources, and cumulative effects through regional, multimedia studies codesigned with communities and Indigenous partners (Figure 1). The ICMP broadly considers known and/or suspect substances (targeted) and unknowns (nontargeted analysis (NTA)) and links mixture compositions and/or sources to biological responses across levels of organization, while using new approach methods (NAMs) to replace, reduce, or refine the use of vertebrate animals.

The ICMP strives to holistically integrate community engagement, monitoring, effects assessment, and management within one design. The path to implementation of ICMP outputs under CEPA is designed to inform risk assessment (RA) substance prioritization, advance cumulative effects assessment methods, and evaluate risk management (RM) options, aligning with CEPA’s modernization.

## ICMP Conceptual Architecture (2023–2027)

1. Problem formulation and prioritization: Identify regions, mixtures, and exposure pathways with a high likelihood of cumulative effects, leveraging existing data (e.g., national pollutant release inventory (NPRI)) and local knowledge; augment by being responsive to stakeholder and Indigenous priorities.
2. Multimedia monitoring and mixture characterization: Collections of both air and air deposition, water, sediment, soil, biota, wastewater, and landfill leachate; perform targeted and NTA to capture known and unknown chemicals and transformation products.
3. Effect-based evaluation (NAMs/NTA): Link mixture profiles to biological responses using toxicogenomics, eDNA, fish/frog/snail embryo tests, histone adduction assays, oxidative potential to detect sublethal and cumulative effects.
4. Source apportionment and transformation products: Combine fingerprinting, cluster analysis, oxidation chamber experiments, and spatiotemporal mapping to attribute mixtures to sectors/facilities and prioritize transformation products for further screening.
5. Inform RA and RM activities: Develop, test, and advance approaches to address chemical mixtures and cumulative effects and provide field-anchored data on chemical mixtures and effects to support RA activities, including prioritization, and RM under CEPA.
6. Engagement and knowledge transfer: Establish data sharing and interpretation agreements with Indigenous partners; publish on Canada.ca and the Open Data Portal; deliver plain language outputs and visualization of data.

## What This Integrated, Holistic Approach Seeks to Deliver

- An evaluation of baseline and drivers of multipollutant airborne mixtures and deposition, including oxidation-derived transformation products; oxidative potential and other biological effects linked to chemicals and chemical classes; visualization tools to depict cumulative exposures and contrasting impacts on populations.
- An evaluation of point and nonpoint source mixture impacts on different trophic levels across laboratory/field gradients; omics/eDNA to detect early ecotoxicological effects and biodiversity shifts; conduct source apportionment where appropriate. As one of the first studies produced by the ICMP,^5^ a national survey of municipal wastewaters found that specific ion exchange resins showed superior performance for NTA in tandem with molecular networking and compound class scoring (CCS). This study identified >800 key contaminants (e.g., pharmaceuticals and recreational drugs), >50 transformation products, and >2000 unique polyoxyethylene congeners, demonstrating excellent potential for effect correlations and source apportionment within ICMP and beyond.
- An evaluation of PFAS and co-occurring mixtures in sentinel bird species with varying diets, including histone adduction and gene expression end points. Assess soil/biota bioaccumulation and microbial indicators of potential landfill leachate effects. Using a newly ICMP-developed NAM based on a histone protein adduction assay, nonenzymatic covalent modifications occurred with calf thymus and human H3.3 histone adduction with selected PFAS.^6^ The histone proteome reactivity in vitro suggests that histone–PFAS adducts could be used as biomarkers of PFAS (mixture) exposure and associated toxicities. In addition to legacy PFAS, nontargeted screening (NTS) identified PFAS not captured in existing targeted methods as part of a Canada-wide federal monitoring program on European starlings and their eggs,^7^ e.g., branched isomers of PFHpS, PFNS, PFDS, the 6:2 polyfluoroalkyl phosphate diester, and sulfate conjugates of the 6:2, 8:2, 10:2, and 12:2 fluorotelomer alcohols. Other ICMP research on PFAS in the avian–terrestrial food web of the Brantford site revealed that invertebrates collected on the active landfill site contain levels of PFOS that are 10 times higher than current existing Federal Environmental Quality Guidelines for PFOS in avian diets (8.2 ng/g of wet weight).
- Data to inform existing RA and RM under CEPA: Information generated through the ICMP could be used to prioritize substance groupings or mixtures for further activities under CEPA. It could also provide information relevant to substances already prioritized for assessment under the CEPA Plan of Priorities or listed on the List of Toxic Substances (Schedule 1) of CEPA.
- Advancement of approaches to address chemical mixtures and cumulative effects: A prototype for an ecological RA tool box for mixtures, tailored to the context under CEPA, will be developed to account for the co-occurrence of chemicals. In addition, environmental models will be developed and improved to predict release patterns and emission sources.
- Locally meaningful engagement and Indigenous Knowledge: Co-design sampling with First Nations Partners, share and agree on interpretations prior to public release, and transfer knowledge and support Indigenous led projects, such as the development of air and water monitoring programs.

With these products, the ICMP strives to demonstrate the value of a holistic, field-anchored mixtures approach that can be scaled nationally to augment Canada’s Chemicals Management Plan beyond single-substance paradigms.
